# Evaluation of the antimicrobial activity and safety of *Rhus vulgaris* (Anacardiaceae) extracts

**DOI:** 10.1186/s12906-020-03063-7

**Published:** 2020-09-10

**Authors:** Angela Mutuku, Lizzy Mwamburi, Lucia Keter, Joyce Ondicho, Richard Korir, James Kuria, Timothy Chemweno, Peter Mwitari

**Affiliations:** 1 Department of Biological Sciences, University of Eldoret, P.O. Box 1125-30100, Eldoret, Kenya; 2 Centre for Traditional Medicine and Drug Research, Kenya Medical Research Institute, P.O. Box 54840-00202, Nairobi, Kenya; 3 Centre for Microbiology Research, Kenya Medical Research Institute, P.O. Box 54840-00202, Nairobi, Kenya

**Keywords:** *Rhus vulgaris*, Antimicrobial, Antifungal, Cytotoxicity, Safety, Acute toxicity, Plant extracts

## Abstract

**Background:**

Medicinal plants have been used in the treatment of various ailments in most developing countries. Oral infections are the most prevalent diseases in man. The Rhus family has been found to have antimicrobial, antimalarial, and anti-inflammatory properties. Few studies have been done on *Rhus vulgaris* Meikle. A study was conducted to determine the effect of *Rhus vulgaris* Meikle stem bark extracts against selected oral pathogenic microorganisms and the safety of the extracts in vitro and in vivo.

**Methods:**

Methanol:dichloromethane (1:1), methanol and aqueous extracts were tested for bacteriostatic and bactericidal effects against Methicillin Resistant *Staphylococcus aureus* (MRSA), *Staphylococcus aureus, Streptococcus mutans* and *Candida albicans*. Cytotoxicity of the active extracts was determined using Vero E6 cell lines while safety was evaluated in mice and rats. Phytochemical screening was performed on the methanol extracts. One-way ANOVA and Tukey’s multiple comparisons tests were performed using IBM SPSS statistics 20.0 for antimicrobial assay and acute toxicity testing. One-way ANOVA and Dunnett’s multiple comparison tests were conducted using GraphPad Prism 8.0 for cytotoxicity assay.

**Results:**

Methanol extract of *Rhus vulgaris* showed significant antimicrobial activity against MRSA (12.00 ± 0.00 mm; *p*-value of < 0.005; Minimum Inhibitory Concentration of 0.391 mg/ml; Minimum Bactericidal Concentration of 1.563 mg/ml). The extract were not cytotoxic at 100 μg/ml which was the highest tested concentration. In acute dermal irritation testing, the methanol extract resulted in mild irritation with erythema and flaking that cleared within 8 days. There were no observable adverse effects from oral administration of the extracts (acute oral toxicity testing) at concentrations of 50 mg/kg, 300 mg/kg and 2000 mg/kg. Tannins, saponins, flavonoids, terpenoids, glycosides, alkaloids and phenols were detected in the methanol extract.

**Conclusions:**

Antimicrobial activity of *R. vulgaris* extracts supports its traditional use as a toothbrush. Cytotoxicity demonstrated by the extracts as well as the mild skin irritation warrants further study before *R. vulgaris* can be recommended for the development of effective and safe mouthwashes.

## Background

Globally, oral diseases are highly prevalent and pose a major public health challenge [1]. Oral microbiota, that cause oral diseases, have developed resistance against some antibacterial agents such as metronidazole, tetracycline, erythromycin, cephalosporin and penicillin [2]. Some antimicrobials also have unfavorable side effects such as toxicity, teeth staining, diarrhea, vomiting and the alteration of the oral cavity normal flora [2]. Chlorhexidine mouth wash for example has been shown to result in teeth staining, oral mucous membrane staining, increased calculus formation, parotid swelling, desquamation of the oral mucosa and urticaria [3]. Other antibacterial agents that also result in unfavorable side effects such as toxicity are amine fluorides, cetylpyridinium and chloride [2]. The development of antimicrobial resistance and side effects caused by the current antimicrobials has resulted in a need for alternatives that are safe, user-friendly and cost-effective [2]. Extracts from some plants have been found to be efficacious against oral diseases. *Aloe barbadensis*, *Ocimum sanctum*, *Acacia nilotica*, *Eucalyptus camaldulensis*, *Hibiscus sabdariffa*, *Psidium guajava*, *Mangifera indica, Rosa indica*, and *Azadirachta indica* Miller have demonstrated inhibitory properties against some dental caries and periodontal pathogens [4]. Their activity is postulated to be through the inhibition of microbial growth and bacterial adhesion in biofilms formed on the tooth surface [5].

*Rhus vulgaris* Meikle (Anacardiaceae) is found in tropical and temperate regions [6]. In Kenya, the stem bark of this plant is used in the treatment of malaria [7]. In Uganda, this plant is used for the treatment of toothaches [8] and it is one of the most commonly used chewing sticks [9]. In Tanzania, *R. vulgaris* leaves are traditionally used for the treatment of dental problems and as a refreshment in herbal teas [10]. In regions surrounding Lake Victoria in East Africa, *R. vulgaris* fruits are used for the treatment of tooth ache, coughing, syphilis, gastrointestinal disorders and for the prevention of infections [11]. The *Rhus* genus has demonstrated significant antimicrobial, antifungal, antiviral, antioxidant, antimalarial and cytotoxic properties [6]. The aqueous extracts of *R. vulgaris* have been reported to possess good antibacterial activity against *Streptococcus mutans* [9]. The dichloromethane, ethyl acetate and aqueous extracts of *R. vulgaris* stem bark, root and leaf have shown antibacterial activity against *Proteus mirabilis*, *Klebsiella oxytoca, Klebsiella pneumoniae, Salmonella typhi, Escherichia coli, Pseudomonas aeruginosa* and *Salmonella kisarawe* with MIC values ranging from 0.39 mg/ml to 25 mg/ml while gentamycin, positive control, exhibited MIC values ranging from 0.003 mg/ml to 0.25 mg/ml [10]. The dichloromethane, ethyl acetate and aqueous extracts of *R. vulgaris* stem bark, root and leaf have also demonstrated antifungal activity against *Candida albicans* and *Cryptococcus neoformans* with MIC values ranging from 1.56 mg/ml to 25 mg/ml while fluconazole, positive control, demonstrated an MIC value of 0.19 mg/ml [12]. The aqueous extracts of *R. vulgaris* (500 mg/ml) stem bark, root and leaf have demonstrated good antioxidant activity of 80.11, 80.25 and 80.62% respectively at 30 min [11]. *R. vulgaris* methanolic extract (1000 mg/kg) showed greater anti-inflammatory activity compared to indomethacin (10 mg/kg), the standard anti-inflammatory drug, with a decrease in inflammation for up to 90 min [13]. The dichloromethane, ethyl acetate and aqueous extracts of *R. vulgaris* stem bark, root and leaf have exhibited moderate to toxic toxicity against brine shrimp with LC_50_ values ranging from 3.55 μg/ml to 734.06 μg/ml while cyclophosphamide, the positive control, demonstrated an LC_50_ value of 15.28 μg/ml [12].

Plants protect themselves from pathogens through the synthesis of secondary metabolites or bioactive molecules known as phytochemicals which exhibit antimicrobial properties [14]. Phytochemical studies on *R. vulgaris* have revealed that it contains terpenoids, flavonoids and terpenes [6]. Bioactive compounds are produced by plants in small concentrations, the extraction method and solvents selected are crucial in the discovery of potential antimicrobial agents [15]. Phytochemical extraction depends on the polarity of the solvents used. A single solvent cannot therefore be expected to extract all the phytochemicals [16]. The use of methanol solvent has led to the extraction of the highest concentration of phenolics, terpenoids, flavonoids and alkaloids [17]. Dichloromethane solvent is useful for the extraction of non-polar compounds while water extracts most of the polar compounds [18]. In the Lake Victoria Basin districts of Uganda, *R. vulgaris* fruits, stem bark and leaves are prepared for consumption using water through decoction, steaming or the leaves and fruits eaten raw for the treatment of toothache, malaria and syphilis [8]. In this study aqueous, methanol and methanol:dichloromethane (1:1) solvents will be utilized for extraction purposes to ensure the extraction of all the phytochemicals enabling a comprehensive evaluation of the antimicrobial properties against selected pathogens and a complete toxicological assessment of *R. vulgaris*.

To the best of our knowledge, after conducting extensive research, no studies have been done on the antibacterial, antifungal and cytotoxic activity of *R. vulgaris* in Kenya. Geographical distribution plays a major role in the phytochemical composition of plants [19], hence, provides a good basis for research into the antibacterial, antifungal and cytotoxic properties of *R. vulgaris* growing in Kenya. Additionally, no studies have been reported on acute toxicity and acute dermal irritation of *R. vulgaris* extracts. The in vivo toxicity evaluation of plant extracts is crucial for the establishment of their safety profiles. Acute toxicity assays provide useful information on the safe and lethal dose range of plant extracts [20].

This study was designed to provide valuable information on the antimicrobial activity and safety of *R. vulgaris* extracts in order to determine its potential use in the maintenance of oral health.

## Methods

### Plant materials

*Rhus vulgaris* Meikle stem bark was collected from Mwala Sub-county, Machakos County (1.3520° S, 37.4503° E) based on its undocumented use in the maintenance of oral hygiene by the local community and the use of its stem bark to cure toothache in Uganda [8]. The plant species was authenticated by a botanist, Mr. Patrick B. Mutiso, Chief technologist at the School of Biological Sciences, University of Nairobi. A voucher specimen (AMM2016/002) deposited at the University of Nairobi Herbarium.

### Bacterial cultures

Clinical isolates of *Staphylococcus aureus,* Methicillin-resistant *S. aureus, Streptococcus mutans* and *Candida albicans* were obtained from Centre for Microbiology Research (CMR), Kenya Medical Research Institute (KEMRI).

### Cell lines

Vero E6 cell lines, sourced from American Type Culture Collections (ATTC® CRL-1586™) and cultured at Centre for Traditional Medicine and Drug Research (CTMDR) KEMRI laboratories, were used in cytotoxicity studies.

### Laboratory animals

Male Wistar rats (150–230 g) were used for the acute dermal irritation/corrosion assay and nulliparous female Swiss albino mice (18 g – 26 g) were used for the acute toxicity assay. The animals were obtained from the KEMRI animal house and were kept under standard room conditions. Pelleted feed (Unga Mice Pencils, Unga Feeds Ltd) and water was made available ad libitum*.* Principles of humane laboratory animal care and use were observed according to the Animal Care and Use Committee (ACUC).

## Extraction

The extraction process was carried out according to Simon et al., [21] with slight modifications. The plant samples were washed, cut into small pieces and left to dry for 2 weeks at room temperature away from direct sunlight. The dried plant material was then finely ground using a grinding mill and stored in brown paper bags at room temperature until use. Both aqueous and organic solvent (methanol and methanol:dichloromethane (1:1)) extraction were carried out.

### Aqueous extraction

Distilled water (400 ml) was added to the ground plant materials (100 g) obtained from *R. vulgaris* and placed in a water bath for 1 h and 30 min at 60 °C. Upon cooling, the aqueous solution was filtered twice using Whatman® No. 1 filter paper. The extracts was then lyophilized using a freeze dryer (Edwards freeze dryer Modulyo) then stored in airtight plastic vials at 4 °C until use.

### Organic solvent extraction

The ground plant material (100 g) was soaked in 300 ml of the solvent (methanol; methanol:dichloromethane (1:1)) for 72 h then and agitated periodically. The mixture was filtered twice using Whatman® No. 1 filter paper. The solvent was then removed under reduced pressure using a rotary evaporator (Büchi Rota vapor R-114). A temperature of 60 °C and 55 °C was applied for the methanol and methanol:dichloromethane extracts respectively. Residual solvent was removed by leaving the extracts in the open. The organic extracts were stored in glass vials at 4 °C.

## Antimicrobial assay

### Disc diffusion assay

The Kirby-Bauer disc diffusion method reported by Thirumurugan [22] was used to determine whether the plant extracts could inhibit the growth of the selected pathogenic bacteria and fungi. Mueller Hinton Agar (OXOID LTD Basingstoke, Hampshire, England) was prepared by dissolving 38 g/l in distilled water. Plates were prepared by pouring 15 ml of molten media into sterile petri plates. The molten media was allowed to solidify for 5 min and the inoculum swabbed uniformly. The culture was then allowed to dry for another 5 min. The same technique was applied using Sabouraud’s dextrose agar for fungal species. Sterile discs were impregnated with 20 μl of 100 mg/ml of the plant extracts, allowed to dry slightly and placed on the surface of cultured agar plates. The negative controls used were distilled water for aqueous extracts and 70% Dimethyl sulfoxide (Sigma) for organic extracts. Standard antibiotic discs of sulfamethoxazole/trimethoprim (23.7/1.25 μg) and fluconazole (2 mg/ml) were used as positive controls for bacteria and fungi respectively. Bacterial and fungal culture plates were then incubated at 37 °C for 24 h and inhibition zones measured in millimeters using a ruler. This assay was performed in duplicate. An indication of significant antibacterial activity was taken to be a growth inhibition zone of 10 mm and above as applied by the CMR laboratories, where the work was undertaken.

### Minimum inhibitory concentration assay

The Minimum Inhibitory Concentration (MIC) test adapted from Thirumurugan [22] was only performed on the plant extracts that showed significant antimicrobial activity in the disc diffusion method i.e. an average zone of inhibition of ≥10 mm. Exactly 20 μl of each plant extract was loaded onto a 96-well titer plate and serially diluted from a concentration of 100 mg/ml to 0.05 mg/ml using sterile tryptic soy broth. Gentamicin (20 μl), the positive control, was serially diluted from a concentration of 14 mg/ml to 0.0068 mg/ml using sterile tryptic soy broth. Each plant extract was placed in two rows of the microtiter plate for serial dilution. Gentamicin was also placed in two rows of the microtiter plate for serial dilution. The first row served as the test while the second row served as the control. Microbial suspension (20 μl) was only added to the first row. A control experiment was carried out simultaneously in the second row using plant extracts/positive control of the same dilutions and no microorganisms. The microtiter plates were incubated at 35 °C with low humidity overnight.

### Minimum bactericidal concentration assay

The Minimum Bactericidal Concentration (MBC) assay adapted from Sánchez et al.*,* [23] was performed by subculturing the samples from each well of the MIC microtitre plates onto a fresh drug-free solid medium using sterile swabs. The petri dishes were then incubated overnight. Lack of visible growth was taken as an indication of the bactericidal ability at the particular concentration of the plant extract. From the results of the MBC and MIC assays, the MBC/MIC ratio was calculated to determine the bactericidal and bacteriostatic properties of active extracts recorded based on Konaté et al., [24] where if the MBC/MIC ratio is ≤4.0, the test substance is considered bactericidal, and when the MBC/MIC ratio is > 4.0 it is considered bacteriostatic.

### Cytotoxicity

The 3-(4,5-dimethylthiazol-2-yl)-2,5-diphenyltetrazolium bromide (MTT, Sigma, USA) assay reported by Radol et al., [25] was used to determine the cytotoxic effects of the plant extracts on Vero cell lines. The cells were cultured in T-75 cell culture flasks and incubated in Eagle’s Minimum Essential Medium (MEM) with 10% fetal bovine serum (FBS) at 37 °C in 5% CO_2_. After a confluent cell sheet was achieved, it was dissociated using trypsin and the cells pooled in a 50 ml tube. MEM (40 ml) was added forming a cellular suspension of which a 100 μl of cell suspension containing 2 × 10^5^ cells was seeded in 96-well microtiter plates. In row H, 150 μl of 100 μg/ml DMSO of the highest test sample concentration in duplicate, was pipetted. DMSO was used to solubilize the organic plant extracts. Serial dilutions were conducted by pipetting 50 μl from wells of row H and adding to wells of row G. Another 50 μl was transferred from row G to wells of row F up to row B discarding the last 50 μl of this row. A threefold dilution was performed from row H to B wells. Incubation was conducted for 48 h at 37 °C in 5% CO_2_ allowing the effect of the test sample on the cells to occur. Cells were observed under the inverted microscope. Into each well, 10 μl of MTT dye was added for the colorimetric determination of viable cells. Cells were further incubated for another 4 h at 37 °C in 5% CO_2_. Media was removed from the wells and 100 μl of DMSO (Sigma) added to solubilize the formazan. Reading of the plates was performed on a scanning multiwell spectrophotometer (Multiskan Ex labsystems) at 562 nm and 620 nm as reference. Doxorubicin (50 μg/ml highest drug concentration) was used as a positive control.

### Acute dermal irritation/corrosion assay

The acute dermal irritation/corrosion test was conducted as recommended by the Organisation for Economic Co-operation and Development (OECD) Guideline 404, 2002 according to Mengiste et al., [26] with modifications from Pinto et al., [27]. Rats were used for this test. The animals were randomly selected, marked and placed in cages 5 days prior to the commencement of the test to facilitate acclimatization. Approximately 24 h before the test began, the rats were restrained and an area of approximately 3 cm^2^ shaved using an electric shaver. Any residual hair was removed using a hair removal cream. Exactly 0.5 g of plant extract in 2 ml of 10% polysorbate 80 (Tween® 80) was applied to the cotton gauze, placed in contact with the animal’s skin and held in place with hypoallergenic tape. One rat was used as the control, to which 10% polysorbate 80 was applied. After 4 h, the test substance/control was removed. All animals were examined for signs of erythema and oedema, and the responses scored at 60 min, and then at 24, 48 and 72 h after removal of the test substance. The initial test was performed on one animal. If a corrosive effect was not observed in the initial test, the negative response was confirmed using two additional animals, each with one patch. The animals were observed for 14 days to record the occurrence and reversibility of any irritant/corrosive effects. They were then euthanized using CO_2_, placed in biohazard bags and incinerated.

### Acute toxicity assay

The acute toxicity assay was conducted in mice as recommended by the OECD Guideline 423,2001 according to Alhaddad et al., [28] with minor modifications. Female, nulliparous, 8 weeks old Swiss albino mice were used. Animals were randomly selected, marked and placed in cages 5 days prior to the commencement of this test to facilitate acclimatization. Three mice were placed into each cage and a single dose of the test substance administered orally via canula. A starting dose level used was 50 mg/kg followed by 300 mg/kg and then 2000 mg/kg body weight. Food was withheld for 2 h before and 30 min after administration of the test substance. Treatment of animals at the next dose was delayed for 24 h to assure the survival of the previously dosed animals. Animals were observed during the first 4 h, and daily thereafter, for a total of 14 days. Clinical signs and mortality cases were recorded. During the 2 week study period, the mice were weighed before oral administration and every seventh day thereafter. On the 14th day after administration of the test substance, the animals were euthanized using CO_2_ and subjected to gross necropsy. All gross pathological changes were recorded for each animal. Afterwards, they were placed in biohazard bags and incinerated.

### Phytochemical screening

Since the methanol extracts were the most active in the bioassays, they were subjected to phytochemical screening to detect secondary metabolites using standard qualitative procedures as performed by Gul et al., [29] for glycosides, Fayaz et al., [30] for alkaloids and Anthoney [31] for tannins, saponins, flavonoids, terpenoids, steroids and phenols. Photographs of the color change and tables were used to document the results.

### Tannins

A 0.5 g sample of the crude extract was put in a test tube and 20 ml of distilled water added to it then heated to boiling. The mixture was then filtered and 1% of FeCl_3_ added to the filtrate and observations made. A brownish green coloration indicated the presence of tannins.

### Saponins

The crude plant extract was mixed with 5 ml of water and vigorously shaken. The formation of stable persistent froth indicated the presence of saponins.

### Flavonoids

A portion of the crude extract was added into a test tube. To this, 5 ml of dilute ammonia and 2 ml of concentrated sulfuric acid were added. The appearance of a yellow color indicated the presence of flavonoids.

### Terpenoids

To the plant extracts, 2 ml of chloroform was added and vigorously shaken and then evaporated to dryness. To this, 2 ml of concentrated sulfuric acid was added and heated for about 2 min. A greyish color indicated the presence of terpenoids.

### Glycosides

Salkowski’s test: The extract of the plant material was mixed with 2 ml of chloroform and then 2 ml of concentrated sulfuric acid added carefully and shaken gently. A reddish brown color indicated the presence of the steroidal (steroidal aglycone) part of glycosides.

### Alkaloids

A few drops of Dragendorff’s reagent was added into the test tube containing crude extracts. The resulting yellow precipitate indicated the presence of alkaloids.

### Steroids

Liebermann Burchard reaction: About 2 g of the extract was put in a test tube and 10 ml of chloroform added, filtered and then 2 ml of the filtrate mixed with 2 ml of a mixture of acetic acid and then concentrated sulfuric acid was added along the side of the test tube. Blue green ring indicated the presence of steroids.

### Phenols

The plant extract (3 ml) was put into a test tube and treated with 1–2 drops of 2% of FeCl_3_. Formation of bluish green coloration indicated the presence of phenols.

## Data analysis

In antimicrobial screening, one-way ANOVA and Tukey’s multiple comparisons tests were performed using IBM SPSS statistics 20.0. The means and the standard deviations were derived from the zones of inhibition to determine statistical significance (*p*-value < 0.05). In cytotoxicity testing, one-way ANOVA and Dunnett’s multiple comparison tests were conducted using GraphPad Prism 8.0. Percentage cell viability was derived from absorbance readings in MS Excel data sheets and used to calculate the half maximal inhibitory concentration (IC_50_) using dose-response curves. Statistical significance (*p*-value < 0.05) was established through the comparison of the percentage cell viability after exposure of the cells to the extracts and the standard reference drug. In acute dermal corrosion testing, photographs were taken to document hair growth rate and any skin reactions. In acute toxicity testing, one-way ANOVA and Tukey’s multiple comparison tests were calculated using IBM SPSS statistics 20.0. Comparisons were made between the sample mean weights and control mean weights to establish statistical significance (*p* ≤ 0.05).

## Results

### Antimicrobial assay

**Fig. 1:**
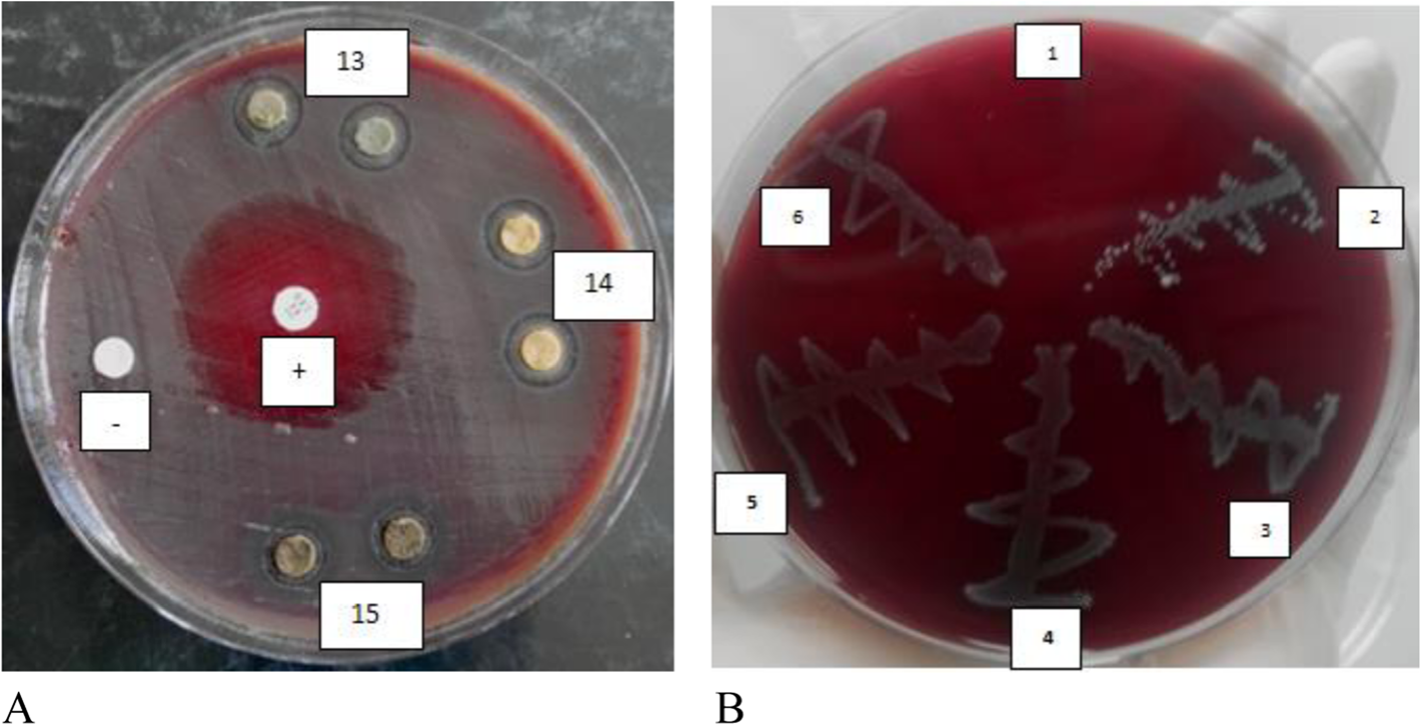
Disc diffusion and MIC plates of *R. vulgaris* extracts against *S. mutans* on Blood agar**. a**: **13:**MeOH extracts, **14:**MeOH:DCM extracts, **15:**Aqueous extracts; **+:**positive control and **-:**negative control. **b**: **1:**MeOH extracts at concentrations of 100 mg/ml, **2:**50 mg/ml, **3:**25 mg/ml, **4:**12.5 mg/ml, **5:**6.25 mg/ml and **6:** 3.125 mg/ml

Table 1 shows the results of the microbial growth inhibition by *R. vulgaris* against MRSA*, S. aureus, S. mutans* and *C. albicans*. The organic extracts of *R. vulgaris* exhibited higher antimicrobial activity compared to the aqueous extracts. Among the organic extracts, methanol extracts demonstrated greater zones of inhibition. The most susceptible microorganism was *S. aureus* followed by MRSA, *S. mutans* and lastly, *C. albicans* with little to no inhibition observed. The MeOH (*p*-value of 0.722) and MeOH:DCM (*p*-value of 0.069) extracts resulted in zones of inhibition of 19.50 mm and 17.00 mm respectively against *S. aureus*. The Methanol (10 mm) and MeOH:DCM (10 mm) *R. vulgaris* extracts gave slightly greater zones of inhibition against *S. mutans* than the aqueous extracts (9 mm), with a *p*-value of < 0.005 (Fig. 1, Plate 1).

**Table 1:** Growth inhibition of microorganisms by *R. vulgaris* extracts

Microorganism	Plant	Plant part	Solvent	Average Zone of inhibition ± Std. dev. of duplicates (mm)	***p***-values
MRSA	*R. vulgaris*	Stem bark	MeOH	12.00 ± 0.00	< 0.005
MRSA	*R. vulgaris*	Stem bark	MeOH:DCM	10.00 ± 0.00	< 0.005
MRSA	*R. vulgaris*	Stem bark	Aqueous	7.00 ± 0.00	0.039
MRSA	Control			6.00 ± 0.00	
*S. aureus*	*R. vulgaris*	Stem bark	MeOH	19.50 ± 0.71	0.722
*S. aureus*	*R. vulgaris*	Stem bark	MeOH:DCM	17.00 ± 2.83	0.069
*S. aureus*	*R. vulgaris*	Stem bark	Aqueous	7.00 ± 0.00	< 0.005
*S. aureus*	Control			24.19 ± 3.60	
*S. mutans*	*R. vulgaris*	Stem bark	MeOH	10.00 ± 0.00	< 0.005
*S. mutans*	*R. vulgaris*	Stem bark	MeOH:DCM	10.00 ± 0.00	< 0.005
*S. mutans*	*R. vulgaris*	Stem bark	Aqueous	9.00 ± 0.00	< 0.005
*S. mutans*	Control			22.13 ± 3.44	
*C. albicans*	*R. vulgaris*	Stem bark	MeOH	6.50 ± 0.00	< 0.005
*C. albicans*	*R. vulgaris*	Stem bark	MeOH:DCM	6.00 ± 0.00	< 0.005
*C. albicans*	*R. vulgaris*	Stem bark	Aqueous	6.50 ± 0.00	< 0.005
*C. albicans*	Control			38.13 ± 4.86	

***MRSA*** Methicillin-resistant *Staphylococcus aureus*, ***S. aureus***: *Staphylococcus aureus*, ***C. albicans***: *Candida albicans*, ***S. mutans***: *Streptococcus mutans*, ***R. vulgaris***: *Rhus vulgaris*, ***MeOH*** Methanol, ***MeOH:DCM*** Methanol:Dichloromethane. The positive control for MRSA, *S. aureus* and *S. mutans* was sulfamethoxazole/trimethoprim (23.7:1.25 μg) standard antimicrobial discs. Plant extract concentration was 100 mg/ml. The positive control for *C. albicans* was fluconazole at a concentration of 2 mg/ml. *p*-values of ≤0.05 demonstrate statistical significance

*R. vulgaris* methanol extracts showed inhibitory and bactericidal activity against MRSA (0.391 mg/ml and 1.563 mg/ml), *S. aureus* (3.125 mg/ml and 3.125 mg/ml) and *S. mutans* (1.563 mg/ml and 100 mg/ml) with MIC and MBC values, respectively. The MeOH:DCM extracts also demonstrated antibacterial properties against MRSA (1.563 mg/ml, 3.125 mg/ml), *S. aureus* (3.125 mg/ml, 12.5 mg/ml) and *S. mutans* (25 mg/ml, 100 mg/ml) with MIC and MBC values, respectively. Gentamicin, the positive control, also demonstrated antibacterial properties against MRSA (0.027 mg/ml, 0.055 mg/ml), *S. aureus* (< 0.014 mg/ml, 0.109 mg/ml) and *S. mutans* (0.007 mg/l, 0.027 mg/ml) with MIC and MBC values, respectively (Table 2). According to Konate et al., [24], if the MBC/MIC ratio is ≤4.0, the substance is considered bactericidal, and if the MBC/MIC ratio is > 4.0, the test substance is considered to be bacteriostatic. Based on the MBC/MIC ratio (Table 2), it was established that the *R. vulgaris* methanol extracts were bactericidal against MRSA and *S. aureus*. *R. vulgaris* methanol extracts were bacteriostatic against *S. mutans*. The MeOH:DCM stem bark extracts were bactericidal against MRSA, *S. aureus* and *S. mutans* while gentamicin, the positive control, was bactericidal against MRSA and *S. mutans* (Fig. 1, Plate 2).

**Table 2:** The MIC and MBC values of active plant extracts

Microorganism	Plant/ test substance	Plant part	Solvent	MIC mg/ml	MBC mg/ml	MBC/MIC Ratio
MRSA	*R. vulgaris*	Stem bark	MeOH	0.391	1.563	4
MRSA	*R. vulgaris*	Stem bark	MeOH:DCM	1.563	3.125	2
MRSA control	Gentamicin			0.027	0.055	2
*S. aureus*	*R. vulgaris*	Stem bark	MeOH	3.125	3.125	1
*S. aureus*	*R. vulgaris*	Stem bark	MeOH:DCM	3.125	12.5	4
*S. aureus* control	Gentamicin			< 0.014	0.109	NA
*S. mutans*	*R. vulgaris*	Stem bark	MeOH	1.563	100	64
*S. mutans*	*R. vulgaris*	Stem bark	MeOH:DCM	25	100	4
*S. mutans* control	Gentamicin			0.007	0.027	4

***NA*** Not applicable, ***MRSA*** Methicillin-resistant *Staphylococcus aureus*, ***S. aureus***: *Staphylococcus aureus*, ***C. albicans***: *Candida albicans*, ***S. mutans***: *Streptococcus mutans*, ***R. vulgaris***: *Rhus vulgaris*, ***MeOH*** Methanol, ***MeOH:DCM*** Methanol:Dichloromethane, The positive control for MRSA, *S. aureus* and *S. mutans* was gentamicin

### Cytotoxicity

**Fig. 2:**
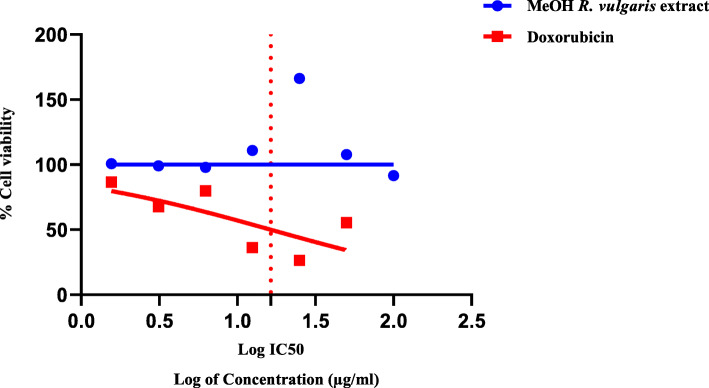
Cytotoxic activities of methanol extract of *R. vulgaris* (SB) and Doxorubicin against Vero cells. *R. vulgaris* methanol extract concentration ranged from 100 μg/ml to 1.5625 μg/ml. Doxorubicin concentration ranged from 50 μg/ml to 0.78125 μg/ml. Doxorubicin exhibited an IC50 value of 16.37 μg/ml

**Fig. 3:**
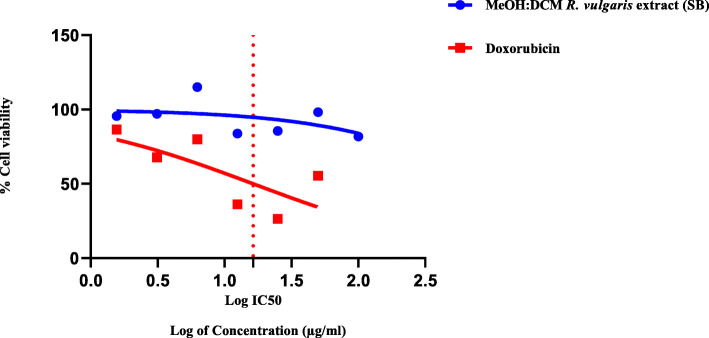
Cytotoxic activities of MeOH:DCM extract of *R. vulgaris* (SB) and Doxorubicin against Vero cells. *R. vulgaris* methanol:dichloromethane extract concentration ranged from 100 μg/ml to 1.5625 μg/ml. Doxorubicin concentration ranged from 50 μg/ml to 0.78125 μg/ml. *R. vulgaris* methanol:dichloromethane extract demonstrated a IC50 value of 1120 μg/ml. Doxorubicin exhibited an IC50 value of 16.37 μg/ml

Figure 2 & Fig. 3 are graphical representations of the percentage cell viability after exposure to different concentrations of *R. vulgaris* extracts and doxorubicin, the standard reference drug.

The MTT assay performed using Vero cells generated IC_50_ values of 1120 μg/ml and 16.37 μg/ml for the MeOH:DCM extracts of *R. vulgaris* and doxorubicin (Fig. 3). The methanol *R. vulgaris* extracts (*p*-value of 0.0039) supported the proliferation of cells and showed no cytotoxic properties in the tested concentrations (Fig. 2). Marginal cytotoxic properties developed as the MeOH:DCM extract (*p*-value of 0.1173) concentration increased (Fig. 3).

### Acute dermal irritation/corrosion

**Fig. 4:**
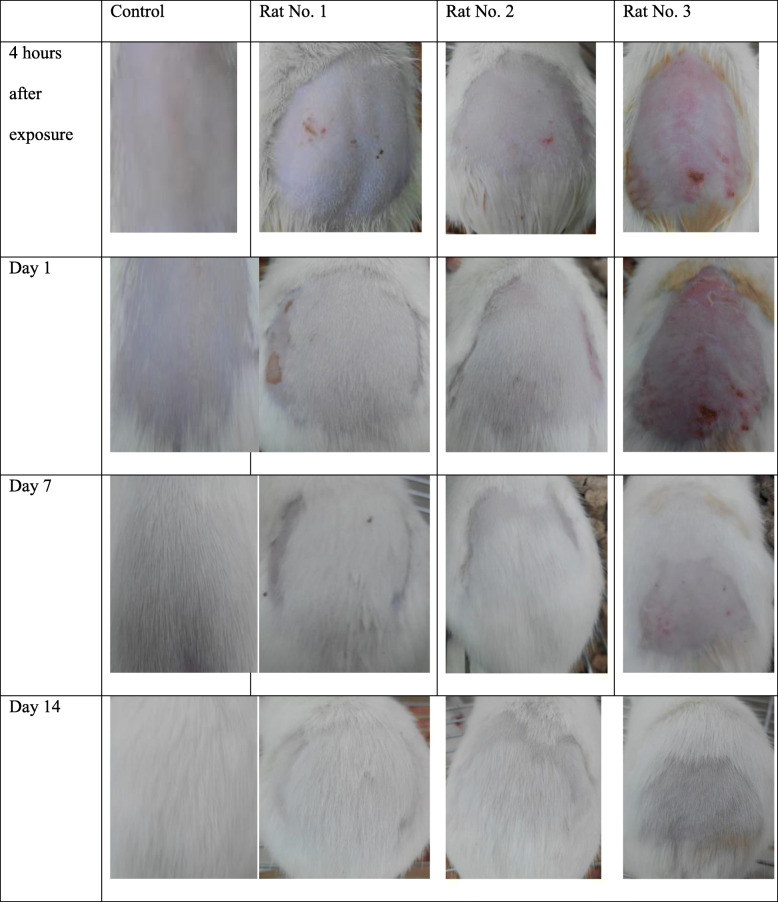
The effects of the exposure of *R. vulgaris* extracts on the depilated skin of Wistar rats

A visual representation of the effects of *R. vulgaris* exposure on the dermis of Wistar rats is displayed in Fig. 4. *R. vulgaris* demonstrated a noteworthy skin reaction in one out of the three rats used. After exposure, the skin of rat one of the rats showed slight erythema after 4 h followed by moderate erythema and flaking of the skin after 24 h. The skin reaction resolved within 8 days.

### Acute oral toxicity

**Fig. 5:**
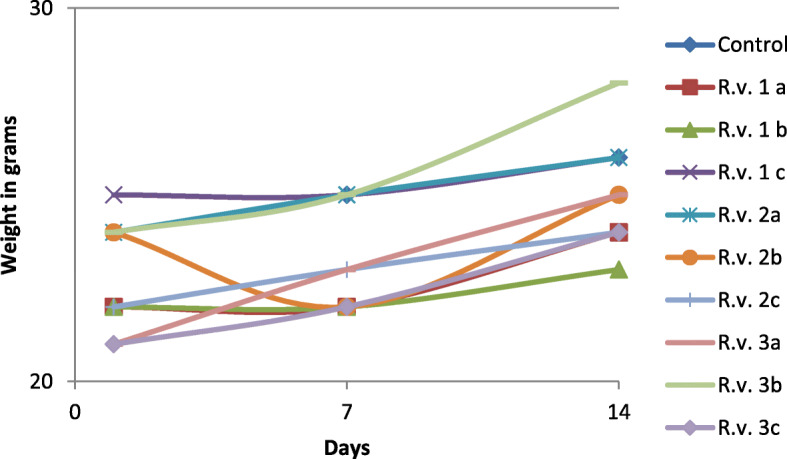
Weight of mice Weights of Mice used in Acute Toxicity assay for Day 1, 7 and 14. *R. vulgaris*: *Rhus vulgaris*, ^a^: Mouse No.1, ^**b**^: Mouse No. 2, ^**c**^: Mouse No. 3, **R.v. 1** ^**a**^: Oral dose of *Rhus vulgaris* (50 mg/kg) administered to Mouse No. 1, **R.v. 1**^**b**^: Oral dose of *R. vulgaris* (50 mg/kg) administered to Mouse No. 2, **R.v. 1**^**c**^: Oral dose of *R. vulgaris* (50 mg/kg) administered to Mouse No. 3, **R.v. 2**^**a**^: Oral dose of *R. vulgaris* (300 mg/kg) administered to Mouse No. 1, **R.v. 2**^**b**^: Oral dose of *R. vulgaris* (300 mg/kg) administered to Mouse No. 2, **R.v. 2**^**b**^: Oral dose of *R. vulgaris* (300 mg/kg) administered to Mouse No. 2, **R.v. 2**^**c**^: Oral dose of *R. vulgaris* (300 mg/kg) administered to Mouse No. 3, **R.v. 3**^**a**^: Oral dose of *R. vulgaris* (2000 mg/kg) administered to Mouse No. 1, **R.v. 3**^**b**^: Oral dose of *R. vulgaris* (2000 mg/kg) administered to Mouse No. 2, **R.v. 3**^**c**^: Oral dose of *R. vulgaris* (2000 mg/kg) administered to Mouse No. 1

The oral administration of *R. vulgaris* at a concentration of 300 mg/kg resulted in hunched posture, inactivity, piloerection and tachypnea in 2 out of 3 mice while at concentrations of 50 mg/kg and 2000 mg/kg no changes in general appearance and behavioral patterns were noted. After 24 h, the general appearance and behavioral patterns of all the mice were normal. The weights recorded on the 1st, 7th and 14th day were not statistically significant (*p*-value > 0.05) when compared to the controls (Table 3, Fig. 5). There was no mortality or gross pathology in any organ at necropsy.

**Table 3:** Weights of Mice used in Acute Toxicity assay for Day 1, 7 and 14

Plant extracts	Concentration	Cage	Day 1	Day 7	Day 14	Mean±	Standard	Significance		
	(mg/kg)						Deviation	Control 1	Control 2	Control 3
Control 1	DH_2_O	C 1^a^	20	20	22	20.6667	1.15470	NA	0.356	1.000
Control 2	DH_2_O	C 2^b^	24	25	26	25.0000	1.00000	0.356	NA	0.983
Control 3	DH_2_O	C 3^c^	20	22	25	22.3333	2.51661	1.000	0.983	NA
*R. vulgaris*	50	R.v. 1^a^	22	22	24	22.6667	1.15470	1.000	0.997	1.000
*R. vulgaris*	50	R.v. 1^b^	22	22	23	22.3333	0.57735	1.000	0.983	1.000
*R. vulgaris*	50	R.v. 1^c^	25	25	26	25.3333	0.57735	0.225	1.000	0.936
*R. vulgaris*	300	R.v. 2^a^	24	25	26	25.0000	1.00000	0.356	1.000	0.983
*R. vulgaris*	300	R.v. 2^b^	24	22	25	23.6667	1.52753	0.936	1.000	1.000
*R. vulgaris*	300	R.v. 2^c^	22	23	24	23.0000	1.00000	0.997	1.000	1.000
*R. vulgaris*	2000	R.v. 3^a^	21	23	25	23.0000	2.00000	0.997	1.000	1.000
*R. vulgaris*	2000	R.v. 3^b^	24	25	28	25.6667	2.08167	0.132	1.000	0.838
*R. vulgaris*	2000	R.v. 3^c^	21	22	24	22.3333	1.52753	1.000	0.983	1.000

There were four cages. One cage had the control group while the other three had the test groups. Each cage had 3 mice. All the mice in one cage received the same concentration of control/plant extract. ***R. vulgaris***: *Rhus vulgaris*, **DH**_**2**_**O**: Distilled water, **C 1**: Control 1, **C 2**: Control 2, **C 3**: Control 3, ^**a**^: Mouse No.1, ^**b**^: Mouse No. 2, ^**c**^: Mouse No. 3, **R.v. 1** ^**a**^: Oral dose of *Rhus vulgaris* (50 mg/kg) administered to Mouse No. 1, **R.v. 1**^**b**^: Oral dose of *R. vulgaris* (50 mg/kg) administered to Mouse No. 2, **R.v. 1**^**c**^: Oral dose of *R. vulgaris* (50 mg/kg) administered to Mouse No. 3, **R.v. 2**^**a**^: Oral dose of *R. vulgaris* (300 mg/kg) administered to Mouse No. 1, **R.v. 2**^**b**^: Oral dose of *R. vulgaris* (300 mg/kg) administered to Mouse No. 2, **R.v. 2**^**b**^: Oral dose of *R. vulgaris* (300 mg/kg) administered to Mouse No. 2, **R.v. 2**^**c**^: Oral dose of *R. vulgaris* (300 mg/kg) administered to Mouse No. 3, **R.v. 3**^**a**^: Oral dose of *R. vulgaris* (2000 mg/kg) administered to Mouse No. 1, **R.v. 3**^**b**^: Oral dose of *R. vulgaris* (2000 mg/kg) administered to Mouse No. 2, **R.v. 3**^**c**^: Oral dose of *R. vulgaris* (2000 mg/kg) administered to Mouse No. 1, ***NA***: Not applicable

### Phytochemical screening

Phytochemical screening was performed to determine the bioactive compounds present in the extract which tested positive for tannins, saponins, flavonoids, terpenoids, glycosides, alkaloids and phenols are shown in Table 4. Steroids were not detected.

**Table 4:** Phytochemical screening of *R. vulgaris* methanol extracts

Phytochemicals	Reagents	Methanol extract
Tannins	Iron (III) chloride	+
Saponins	Frothing test	+
Flavonoids	Ammonia, Sulfuric acid	+
Terpenoids	Chloroform, sulfuric acid	+
Glycosides	Chloroform, sulfuric acid	+
Alkaloids	Dragendorff’s	+
Steroids	Chloroform, acetic acid, sulfuric acid	–
Phenols	Iron (II) chloride	+

**+**: Present, **−**: Absent

## Discussion

In the world millions of people are afflicted by oral diseases [32] through biofilm development [33]. If preventive or curative measures are not undertaken against oral pathogens, systemic or chronic diseases such as diabetes, osteoporosis, rheumatoid arthritis and coronary heart disease may result [33]. The number of oral bacteria in healthy individuals is in the range 10^2^–10^3^, 10^4^–10^8^ in a gingival state and 10^5^–10^8^ in a periodontal state [34].

The tested *R. vulgaris* extracts demonstrated significant antimicrobial activity against some pathogens that cause oral infections. The methanol extract showed the highest activity followed by the MeOH:DCM extract and lastly, the aqueous extract with the least activity. The methanol (12 mm) and MeOH:DCM (10 mm) extracts of *R. vulgaris* demonstrated the greatest growth inhibition against MRSA with a *p* value of < 0.005. Furthermore, the methanol and MeOH:DCM *R. vulgaris* extracts also showed antibacterial activity against *S. mutans* with a mean diameter zone of inhibition of 10 mm. Very slight anti-streptococcal activity was demonstrated by the aqueous extracts (7 mm). Different results were obtained by Odongo et al., [9]. The authors reported that aqueous extracts of *R. vulgaris* showed significant anti-streptococcal activity (24 mm). The inactivity recorded in this study compared to its significant activity in the study by Odongo et al., [9] may be due to variations in phytochemical concentrations as a result of differences in plant factors e.g. age at harvesting as well as other ecological factors [35].

Traditional medicine has been applied in combating antimicrobial resistance [36]. For instance, the stem bark of *R. vulgaris* is traditionally used in the treatment of toothaches [8] supporting its activity against *S. mutans*. Furthermore, extracts of *R. vulgaris* were bactericidal against MRSA and *S. aureus*. MeOH:DCM extracts of *R. vulgaris* were bactericidal while methanol extracts were bacteriostatic against *S. mutans.* The MIC and MBC values of *R. vulgaris* methanol extracts were ≤ 3.13 mg/ml against *S. aureus* and MRSA.

In cytotoxicity testing, MeOH:DCM extracts of *R. vulgaris* exhibited marginal inhibition of proliferation against Vero cells while the methanol extracts supported cellular proliferation. Application of a single oral dose of *R. vulgaris* up to 2000 mg/kg resulted in no observable adverse effects in the test subjects. Further, no manifestations of toxicity were detected during gross pathology. As far as we know, there are no previous studies on the acute toxicity testing of *R. vulgaris* extracts in mice. The observations from this study, in addition to the history of traditional use are an indication of the safety of *R. vulgaris*. Moreover, dermal application of *R. vulgaris* (SB) methanol extracts resulted in mild erythema and scaling. Reversibility of the skin reactions was achieved by the 8th day of the study. Dermal application of the extract additionally resulted in a slower rate of hair regrowth. Similarly, there are no prior reports on the dermal safety of *R. vulgaris* in laboratory animals.

Natural phytochemicals derived from plants provide an affordable and safer means of treating oral diseases [2]. The use of secondary metabolites isolated from medicinal plants in the development of drugs has helped in the fight against microbial infections [36]. Alkaloids, glycosides, saponins, terpenoids, phenols, tannins and flavonoids were present in *R. vulgaris* extracts while steroids and anthraquinones were not detected. Rayne & Mazza [6] also detected similar phytochemicals. Methanol extracts of *R. vulgaris* demonstrated significant inhibitory and bacteriostatic activity against *S. mutans* supporting its traditional use in the treatment of toothache. Further investigations on the cause of delayed hair re-growth after application of the *R. vulgaris* methanol extracts need to be conducted. The modern use of antibacterial agents with harmful side effects has resulted in a significant need for the development of alternatives which are safe and cost-effective [2]. The use of medicinal plants in drug production is preferable as they are biodegradable, affordable to produce and readily available [37].

## Conclusions

The antimicrobial potential demonstrated by *R. vulgaris* extracts supports its traditional use as a toothbrush. However, the cytotoxicity demonstrated by the extracts as well as the mild irritation needs further study before *R. vulgaris* can be recommended for the development of effective and safe mouthwashes.

## Data Availability

All data generated or analysed during this study are included in this published article.

## Abbreviations

*C. albicans*: *Candida albicans*
CO_2_: Carbon (IV) oxide
CMR: Centre for microbiology research
DMSO: Dimethyl sulfoxide
FBS: Fetal bovine serum
FeCl_2_: Iron (II) oxide
FeCl_3_: Iron (III) oxide
MBC: Minimum bactericidal concentration
MEM: Minimum essential medium
MeOH: Methanol
DCM: Dichloromethane
MIC: Minimum Inhibitory concentration
OECD: Organisation for Economic co-operation and development
MIC/MBC: Minimum inhibition concentration/ minimum bactericidal concentration
MRSA: Methicillin-resistant *Staphylococcus aureus*
MTT: 3-(4,5-Dimethylthiazol-2-Yl)-2,5-diphenyltetrazolium bromide
*R. vulgaris*: *Rhus vulgaris*
*S. aureus*: *Staphylococcus aureus*
*S. mutans*: *Streptococcus mutans*
